# Chromatin structural changes around satellite repeats on the female sex chromosome in *Schistosoma mansoni *and their possible role in sex chromosome emergence

**DOI:** 10.1186/gb-2012-13-2-r14

**Published:** 2012-02-29

**Authors:** Julie MJ Lepesant, Céline Cosseau, Jérome Boissier, Michael Freitag, Julien Portela, Déborah Climent, Cécile Perrin, Adhemar Zerlotini, Christoph Grunau

**Affiliations:** 1 Université de Perpignan Via Domitia, CNRS, UMR 5244 Ecologie et Evolution des Interactions (2EI), 52 Avenue Paul Alduy, 66860 Perpignan Cedex, France; 2 Department of Biochemistry and Biophysics, ALS 2011, Oregon State University, Corvallis, OR 97331-7305, USA; 3 CEBio - Centro de Excelência em Bioinformática, Rua Araguari, 741/301 - Barro Preto - BH/MG - CEP 30190-110, Brazil

## Abstract

**Background:**

In the leuphotrochozoan parasitic platyhelminth *Schistosoma mansoni*, male individuals are homogametic (ZZ) whereas females are heterogametic (ZW). To elucidate the mechanisms that led to the emergence of sex chromosomes, we compared the genomic sequence and the chromatin structure of male and female individuals. As for many eukaryotes, the lower estimate for the repeat content is 40%, with an unknown proportion of domesticated repeats. We used massive sequencing to *de novo *assemble all repeats, and identify unambiguously Z-specific, W-specific and pseudoautosomal regions of the *S. mansoni *sex chromosomes.

**Results:**

We show that 70 to 90% of *S. mansoni *W and Z are pseudoautosomal. No female-specific gene could be identified. Instead, the W-specific region is composed almost entirely of 36 satellite repeat families, of which 33 were previously unknown. Transcription and chromatin status of female-specific repeats are stage-specific: for those repeats that are transcribed, transcription is restricted to the larval stages lacking sexual dimorphism. In contrast, in the sexually dimorphic adult stage of the life cycle, no transcription occurs. In addition, the euchromatic character of histone modifications around the W-specific repeats decreases during the life cycle. Recombination repression occurs in this region even if homologous sequences are present on both the Z and W chromosomes.

**Conclusion:**

Our study provides for the first time evidence for the hypothesis that, at least in organisms with a ZW type of sex chromosomes, repeat-induced chromatin structure changes could indeed be the initial event in sex chromosome emergence.

## Background

The origin and evolution of sexuality is one of the most fascinating topics in evolutionary biology. Sex can be determined by several mechanisms, such as environmental stimuli (environmental sex determination) or genetic differences between males and females (genetic sex determination). Genetic sex determination is mainly based on the acquisition of sex chromosomes, a more stable strategy than environmental determinism, especially when the environment becomes variable. The principle steps leading to the emergence and evolution of sex chromosomes have been proposed by Charlesworth *et al*. [1] and Rice [2]. In this model, the emergence of a locus with female fertility and male sterility and another locus with male fertility and female sterility led to the establishment of a small sex-determining region on ordinary autosomes in hermaphrodite ancestors. These so-called proto-sex chromosomes are hardly distinguishable. To prevent the production of infertile individuals, recombination of these loci becomes restricted [3,4]. This crucial step is intensively debated and two mechanisms of action have been proposed: (i) structural changes by translocation or inversion (reviewed in [5]); or (ii) chromatin status changes involving heterochromatization of the heterosexual chromosome [4,6-9]. Heterochromatization of the sex-determining region has been shown in species with primitive or nascent sex chromosomes, such as in papaya or tilapia (reviewed in [10]). The suppression of recombination between the heterochromosome and its homologue would trigger gradual degradation of the heterochromosome (Y in XY systems, or W in WZ systems) because genes that are not essential for males (in XY systems) or females (in WZ systems) show accelerated rates of mutation and deletion. Consequently, the heterochromosome becomes progressively gene-poor (for example, [11]) and in the extreme case the degradation process can lead to the complete loss of the heterochromosome (for example, [12]).

We decided to investigate the role of chromatin structural changes in sex chromosome emergence by using a basal metazoan species harboring a ZW system, the acoelomate *Schistosoma mansoni*. Schistosomes are parasitic plathyhelminthes that are responsible for schistosomiasis (bilharziosis), an important parasitic human disease ranking second only to malaria in terms of parasite-induced human morbidity and mortality [13]. *S. mansoni*'s life cycle is characterized by passage through two obligatory hosts: the fresh-water snail *Biomphalaria glabrata *(or other *Biomphalaria *species, dependent on the geographical location), for the asexual stage; and human or rodents for the sexual adult stage. The sex of the parasite is determined in the eggs (syngamic determination). Eggs are excreted with the host feces and free-swimming larvae (miracidia) are released when the eggs come into contact with water. These miracidiae infect the freshwater mollusk host and transform into primary and secondary sporocysts. Finally, a third larval stage, the cercariae, capable of infecting the vertebrate host, is released into the water. Once in the human or rodent host, morphological differences between female and male adults develop, and these then mate and produce eggs. In the larval stages, schistosome males and females are genetically different but morphologically identical; the sexual dimorphism (that is, the phenotypic expression of sex differentiation) is restricted to the adult stage. All stages are experimentally accessible, which allows the study of chromatin structural modifications for all stages of the life cycle.

Analysis of metaphase spreads indicates that sex is determined in schistosomes by sex chromosomes, with female being the heterogametic sex (ZW) and male the homogametic sex (ZZ) [14]. In some schistosoma species, there is a clear size difference between W and Z, while in other species, such as *S. mansoni*, discrimination is solely based on chromatin structure [15]. This makes *S. mansoni *a model of choice to study the involvement of chromatin structural changes in sex determination of a model harboring a ZW system. In addition, and in contrast with most other plathyhelminth species, schistosomes are gonochoric [16]. This suggests that, in general, being hermaphrodite is an advantage in this phylum, probably through minimizing the risk that is associated with finding a mate inside the host [17]. In Schistosomatidae, the acquisition of separated sexes was concomitant with the invasion of warm-blooded animals [16]. This could be explained by the benefit that genetic diversity provides against the sophisticated immune system of warm-blooded vertebrate hosts and/or by the specialization of each gender for a limited set of 'domestic tasks' [16,18,19]. This particular feature of schistosomes in the plathyhelminth phylum provides the opportunity to study sex chromosome emergence.

The genome of *S. mansoni *was sequenced and initially only partially assembled (version 3.1 with 19,022 scaffolds) [20]. During the preparation of this manuscript, an improved version with assembly at the chromosome level became available (version 5.2 with 882 scaffolds) [21], and Criscione *et al*. [22] constructed a linkage map for 210 version 3.1 scaffolds using microsatellite markers. They identified eight linkage groups corresponding to the seven autosomes and one sex chromosome [22], indicating that the sex chromosomes recombine. Nevertheless, Criscione *et al*. discovered a small region of roughly 18 Mb on the sex chromosome that shows recombination repression. Several open questions remain to be answered. First, it is not clear what are the genetic differences between W and Z chromosomes of *S. mansoni*, or in other words, what are the W- and what are the Z-specific sequences. Second, the mechanism of recombination repression between *S. mansoni *sex chromosomes is not clear. As outlined above, either inversion events or heterochromatization [7,9,23] have been proposed for other species. The specific objectives of the present study were to determine what the sex-specific DNA sequences of *S. mansoni *are, and how heterochromatization of the W chromosome might be initiated. We present here evidence that *S. mansoni *sex chromosomes contain large pseudoautosomal regions. Outside these regions, Z-specific sequences are composed of unique sequences and interspersed repeats. W-specific sequences are almost entirely composed of satellite-type repeats located in the heterochromatic region of the W chromosome. While no female-specific gene could be identified, many of the female repeats are transcribed in the larval stages of the parasite but never in the adults. This loss of transcriptional activity and the development into adults is accompanied by chromatin structural changes around the W-specific repeats. We develop a model in which female-specific repeats are expressed to induce a change in chromatin structure of the W chromosome specifically in the sexual part of the life cycle, leading to functional heterogametism.

## Results

### The *S. mansoni *sex chromosomes Z and W share large pseudoautosomal regions

We had previously sequenced genomic DNA of female and male *S. mansoni *individuals of the DFO strain using Illumina sequencing (National Center for Biotechnology Information Sequence Read Archive (NCBI SRA) submission number SRA012151). We aligned the 8,600,198 sequences from the male samples and the 9,355,380 sequences from the female samples to the 19,022 known scaffolds of the *S. mansoni *genome assembly using SOAP. We then calculated for each scaffold the ratio between sequences that match with the scaffold in question ('hit') for the male and the female DNA. The rationale behind this approach was that, in males (ZZ), Z-specific scaffolds should show two times higher hit counts than in females (WZ). We searched for scaffolds with at least 10 hits per 1 kb in the female and the male genome, at least 10 kb in length, and a male/female hit-count ratio ≥1.68. Using these parameters we identified 15 scaffolds spanning 6,436,718 bp (roughly 10% of the estimated size of the sex chromosomes [22]). We consider these scaffolds (Smp_scaff000398, Smp_scaff018906, Smp_scaff000301, Smp_scaff001995, Smp_scaff000218, Smp_scaff000465, Smp_scaff000514, Smp_scaff000425, Smp_scaff001883, Smp_scaff001948, Smp_scaff000059, Smp_scaff000044, Smp_scaff000576, Smp_scaff000019, Smp_scaff018900) to be specific for the Z chromosome. We confirmed these *in silico *results for representative regions in a subset of 13 arbitrarily chosen scaffolds (5 Z-specific and 8 pseudoautosomal) by quantitative PCR (qPCR; Table 1). With the exception of one scaffold (Smp_scaff000120), qPCR confirmed next generation sequencing hit-count ratios. When the working draft of the fully assembled sequence W/Z chromosome became available [21], we repeated the SOAP alignment. In this new assembly, Smp_scaff000019 was placed on chromosome 2. We showed before [24] that at least 105 scaffolds (436,269 bp) were specific for the W chromosome in females (male DNA did not align to these scaffolds). In conclusion, in genome assembly version 3.1 more than 90% of the non-repetitive part of the Z chromosome and the W chromosome are identical (pseudoautosomal). In version 5.2, the pseudoautosomal region spans 70% of the assembled W/Z chromosome.

**Table 1:** Comparison of the ratio of relative amounts of genomic DNA in male and female adults of *S. mansoni *

	Male (ZZ)/female (WZ)	
	
Scaffold	NGS hit-count ratio	qPCR ratio
Smp_scaff000059	1.76	2.00 ± 0.15
Smp_scaff000425	1.82	1.76 ± 0.11 (region 1)
Smp_scaff000425	1.82	1.83 ± 0.11 (region 2)
Smp_scaff000012	1.3	1.61 ± 0.03
Smp_scaff000047	1.06	0.87 ± 0.04 (region 1)
Smp_scaff000047	1.06	0.87 ± 0.12 (region 2)
Smp_scaff000074	1.02	0.87 ± 0.06 (region 1)
Smp_scaff000074	1.02	0.99 ± 0.13 (region 2)
Smp_scaff000019	1.69	1.76 ± 0.07
Smp_scaff000120	1.61	1.10 ± 0.08
Smp_scaff000054	0.95	1.04 ± 0.09
Smp_scaff000034	0.94	1.07 ± 0.13
Smp_scaff000050	0.93	0.99 ± 0.21
Smp_scaff000024	0.93	0.95 ± 0.09
Smp_scaff000252	0.95	0.93 ± 0.03
Smp_scaff000264	0.96	0.93 ± 0.02

Relative amounts of genomic DNA in male and female adults of *S. mansoni *were measured by next generation sequencing (NGS) hit counts or qPCR in 13 scaffolds (3 scaffolds were sampled in the 2 different regions, 'region 1' and 'region 2').

### The Z-specific region of the Z chromosome is composed of unique sequences and interspersed repeats

The region that is covered by the 15 Z-specific scaffolds contains 205 putative genes (according to the gene predictions in SchistoDB). For 118 genes, a function could be predicted based on sequence similarities (Additional file 1). Among those there are at least four genes that code for proteins that are predicted to be involved in spermatogenesis or for which paralogous genes show testis-specific expression. Nevertheless, for the moment it cannot be concluded that these genes are involved in sex differentiation and further analysis is necessary to clarify the role of these genes. Interspersed repeats were also observed in this genomic region but none of them are Z-specific. The Z-specific region in assembly 3.1 is 6.5 Mb in size. In assembly version 5.2 it spans about 18 Mb and, according to [21], contains 782 genes.

### A region on the sex chromosomes with repressed recombination contains Z-specific sequences but also pseudoautosomal sequences

**Figure 1:**
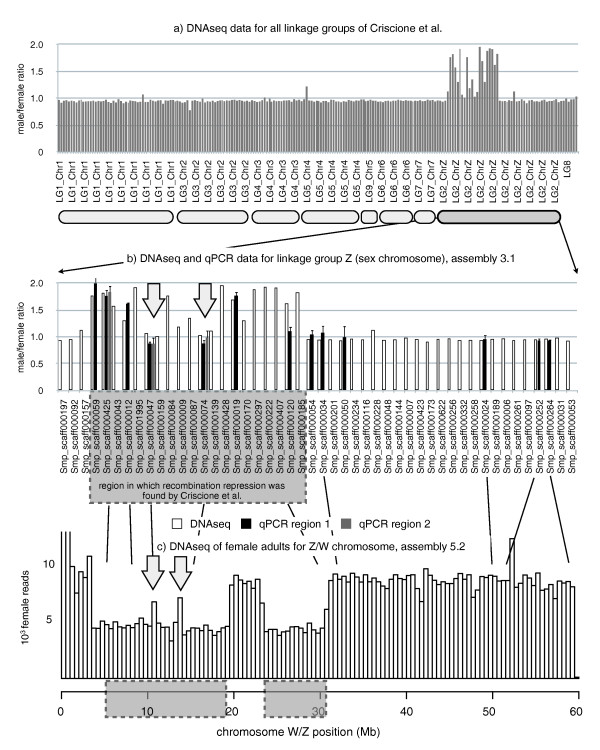
**Next generation sequencing hit-count ratios between male and female genomes**. **(a) **All scaffolds arranged by Criscione *et al*. [22] in linkage groups. For autosomes, the expected ratio would be 1; for Z-specific scaffolds, the expected ratio would be 2 . Scaffolds are in the order defined by Criscione *et al*. **(b) **Male/female hit-count ratios and qPCR ratios for the Criscione *et al*. linkage group 2 (sex chromosomes). **(c) **Profile of hit counts along the latest version of the sex chromosome [21] for DNA extracted from females. One bar corresponds to 0.5 Mb. Arrows indicate the two pseudoautosomal blocks within the region where recombination is repressed.

Having identified pseudoautosomal scaffolds and Z- and W-specific sequences, we searched for the location of these sequences on the chromosomes. For the Z-specific scaffolds we explored an existing linkage map for the sex chromosomes [22]. The results are represented in Figure 1. All mapped Z-specific scaffolds are located in a region of the Z-chromosome for which repression of recombination was described. However, this region also contains pseudoautosomal scaffolds with a hit-count ratio of around 1. Consequently, recombination repression in this region is not due only to absence of sister chromatid sequences. This result was confirmed with assembly version 5.2. In this assembly, a block of sequences originally identified as linkage group 8 [22] was inserted at position 20 to 25 Mb. Consequently, this region recombines, but two smaller regions at 12 to 15 Mb are homologous on the Z and W chromosome but recombination repression occurs (Figure 1).

### The female-specific region of the W chromosome is composed of repetitive sequences

As mentioned above, in an earlier publication we had shown that at least 105 scaffolds (436,269 bp) are specific for the W chromosome in females. We had also indications that a large part of female-specific sequences are composed of repetitive sequences because they matched to known repeats in a repeat database. Nevertheless, 15 to 19% of the massive sequencing data did not correspond to any of the known scaffolds and repeats [24]. These results suggested that they might relate, at least in part, to unknown repetitive sequences. We therefore *de novo *assembled all massive sequencing reads that did not match with unique sequences in the *S. mansoni *genome. SOAP was used to remove *in silico *all female and male reads that correspond to unique sequences, and velvet in combination with a commercial long read assembler was used to assemble the remaining sequences into 8,594 individual repeat contigs (minimum length 80 bp; maximum length 2,169 bp; average length 168 bp). The minimum length corresponds to the used velvet parameter. We then applied our earlier described whole-genome *in silico *subtractive hybridization (WISH) approach [24] to identify female-specific repeats. Thirty-three new repeat sequences were identified to be specific for the female W chromosome, giving a total of 36 W-specific repeats (combined literature data and our data). Several *in silico *methods were used to classify the repeats and their specificity was confirmed by PCR on male and female individuals (abundant in females, very weak signal or absence of amplification in males). The results are summarized in Table 2. Three repeats were already known, 33 repeats are new. The size of the consensus sequence for each assembled repeat was confirmed by PCR on female and male individuals (Additional files 2 and 3). EST data and RT-PCR show that at least eight repeats are transcribed. For a subset, copy number was estimated by qPCR and is moderate (100 to 400 copies), with the exception of SMAlphafem-1 (several thousand copies, confirming earlier estimations [25]). The copy number was estimated using quantitative DNA with a unique W-specific region on scaffold Smp_scaff018821 as reference (positions 2,194 to 2,312).

**Table 2:** W-chromosome-specific repeats of *S. mansoni *

GenBank accession number	Name	Length (bp)	Percentage female hits	Repeat family^a^	Transcription evidence	FISH localization	Copy number estimate^b^	Reference
U12442	SMAlphafem-1	338	99.86	SMAlpha retroposon	RT-PCR	p-arm	60,000 - 70,000	[61,62]
J04665	W1	482	100	Retro		Middle of q-arm at frontier between heterochromatin and euchromatin as satellite, middle of q-arm	500	[26,27]
U10109	W2	715	100				400	[62]
HQ880214	W3	786	100	LTR, highly similar to W2, highly similar to R = 407	No transcription (RT-PCR)	As satellite in the middle of q-arm at frontier between heterochromatin and euchromatin or also in the pericentromeric region	200	
HQ880209	W4	1132	99.52	Highly similar to R = 879	RT-PCR	Same location as W1	800	
HQ880217	W5	1129	99.27	LTR, similar to Perere-2, identical to R = 564	EST and RT-PCR	Either at the frontier of heterochromatin and euchromatin of the q-arm or in the pericentromeric region, or at both locations		
HQ880215	W6	310	99.88	Retro	EST	In the pericentromeric region		
HQ880210	W7	1000	100	DNA transposon, 97% identical to GenBank accession number XP_002570219 (hypothetical protein Smp_186230)		In the pericentromeric region		
HQ880218	W8	266	99.97	Tandem repeat (previously described as TR266), DNA transposon		Either at the frontier of heterochromatin and euchromatin of the q-arm or in the pericentromeric region		
HQ880211	W9	803	100	LINE2, similar to SjR2 retrotransposon	EST			
HQ880212	W10	682	100	LTR				
HQ880213	W11	376	100	LINE, similar to R = 170				
HQ880216	W12	264	100	Retro, 97 to 100% identical to several hypothetical S.m. proteins				
HQ880219	W13	258	100	Retro		In the middle of the heterochromatic part of the q-arm as satellite		
HQ880220	W14	209	100	DNA transposon, similar to R = 170				
HQ880221	W15	185	96.62	DNA transposon				
HQ880222	W16	164	100	Similar to R = 116				
HQ880223	W17	160	100	LTR				
HQ880224	W18	160	100	LTR	EST			
HQ880225	W19	139	100	Retro, 100% identical to GenBank accession number XP_002569391 (hypothetical protein Smp_181820)	EST			
HQ880226	W20	138	100					
HQ880227	W21	138	99.84	DNA transposon, similar to R = 116				
HQ880228	W22	132	100	DNA transposon				
HQ880229	W23	125	100	DNA transposon				
HQ880230	W24	115	99.56	Retro, similar to Sh122 repeat and R = 31				
HQ880231	W25	112	99.65	LTR				
HQ880232	W26	111	100	DNA transposon				
HQ880233	W27	110	100	DNA transposon, similar to R = 133 and Sh microsatellite C2				
HQ880234	W28	108	100	Similar to Sh microsatellite C140				
HQ880235	W29	97	100	Similar to Sb Sbov20 repeat				
HQ880236	W30	96	99.05	LTR				
HQ880237	W31	92	100	LTR				
HQ880238	W32	89	96.88	DNA transposon				
HQ880239	W33	86	99.86	LTR				
HQ880240	W34	82	100	DNA transposon				
HQ880241	W35	80	100	Retro	EST			

Because of the WZ-type chromosome set of females, these repeats are female-specific. ^a^Combined Censor, Blast, Teclass results. ^b^qPCR based. LINE, long interspersed element; LTR, long terminal repeat.

We used SchistoDB to identify genes that could be located within the region that is spanned by the repeats. Eight putative genes were identified in the vicinity of the repeats (not more than 5 kb away). Manual inspection of all loci showed that female next generation sequencing hits can be found for four putative genes, and male hits are absent (Smp_186230, Smp_190410, Smp_117150, Smp_117160). However, three genes (Smp_190410, Smp_117150, and Smp_117160) are identical and the predicted coding regions are small (243 bp for Smp_190410, 327 bp for Smp_186230). No significant similarity to known proteins could be found with blastx. Blast against the genome shows that these putative genes are not unique and it remains to be answered whether these sequences are actually transcribed and code proteins.

### Female-specific repeats are arranged as large satellite type blocks in the heterochromatic region of chromosome W

**Figure 2:**
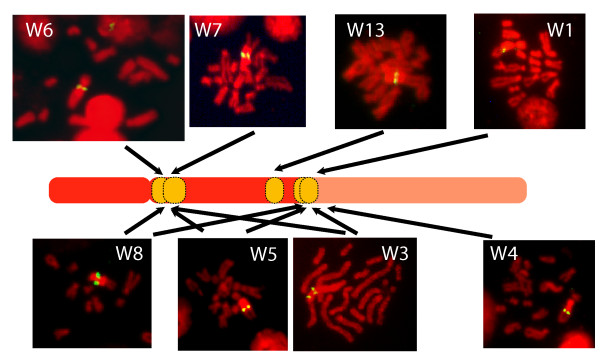
**FISH results with representative pictures of metaphase spreads (chromosomes stained with propidium iodide, probes labeled with fluorescein isothiocyanate, pictures taken separately, colorized and overlaid)**. The positions are indicated on a schematic representation of the W chromosome. The heterochromatic region of the chromosome is in dark red.

To identify the localization of the most abundant female-specific repeats, W1, W3-8 and W13, we used fluorescent *in situ *hybridization (FISH) on late secondary sporocyst metaphases (Figure 2). All studied repeats are (i) arranged as large satellite blocks and (ii) localized in the heterochromatic region of the W chromosome (darker propidium iodide staining), either in the pericentromeric region or on the euchromatin/heterochromatin boundary of the long arm. None of the tested repeats was found on the short arm of chromosome W. Repeats W6 and W7 are specific for the pericentromeric region of the q-arm, and W1 and W4 are located on the frontier of the heterochromatic region. W1 was already known [26] and we confirm the earlier FISH results that localized it to the distal part of the heterochromatic region of Wq [27]. Hirai *et al*. [27] described a euchromatic gap region (eg3) in the vicinity of the W1 chromosome. We did not see this gap, which might be due to the lower resolution of our equipment or differences between the used *S. mansoni *strains. W1 shows genetic instability and in some cases was also found in males [28]. The reason for this could be the close proximity to euchromatin and one might expect such a behavior also for W4. W3, W5 and W8 can be found in both the pericentromeric and the frontier region. W13 is localized roughly in the middle of the heterochromatic part of the q-arm. Results are summarized in Table 2. Copy number estimates (Table 2) correspond to what was found in the literature: 500 to 1,000 copies per genome for W1 [26] and 20,000 to 200,000 for SMAlphafem-1 [25].

### Several of the female-specific repeats are transcribed in larvae but not in adults

**Figure 3:**
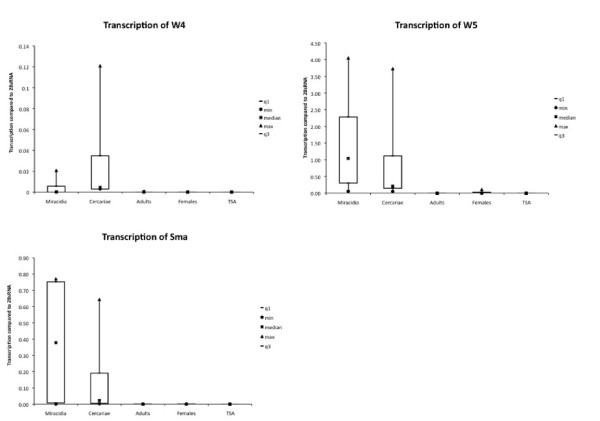
**Transcription levels for repetitive sequences SMAlphafem-1 (Sma), W4 and W5**. Transcription was calculated as a ratio to 28S RNA. Experiments were repeated three to five times for each sample. Maximum, triangle; minimum, circle; quantil 2 to 4, large rectangle.

EST data suggested that some of the repeats could be transcribed and transcription of W1 and SMAlphafem-1 was described for cercaria [29]. We extracted RNA from different life cycle stages and quantified the transcription level for repeats W3, W4, W5 and SMAlphafem-1. For repeats W3 we did not find significant transcription above background; however, repeats W4, W5 and SMAlphafem-1 are transcribed in the larval stages. No transcripts could be detected in adult couples or immature females (Figure 3). At the genomic DNA level, we observed ≤20% differences in repeat copy numbers (measured by qPCR) between different biological samples, but we did not observe a decrease in copy number, that is, shrinking of repeats, during the life cycle. Absence of transcription in adults is not, therefore, due to absence of repeats in the genome.

### The chromatin structure around the female-specific repeats changes during the life cycle

**Figure 4:**
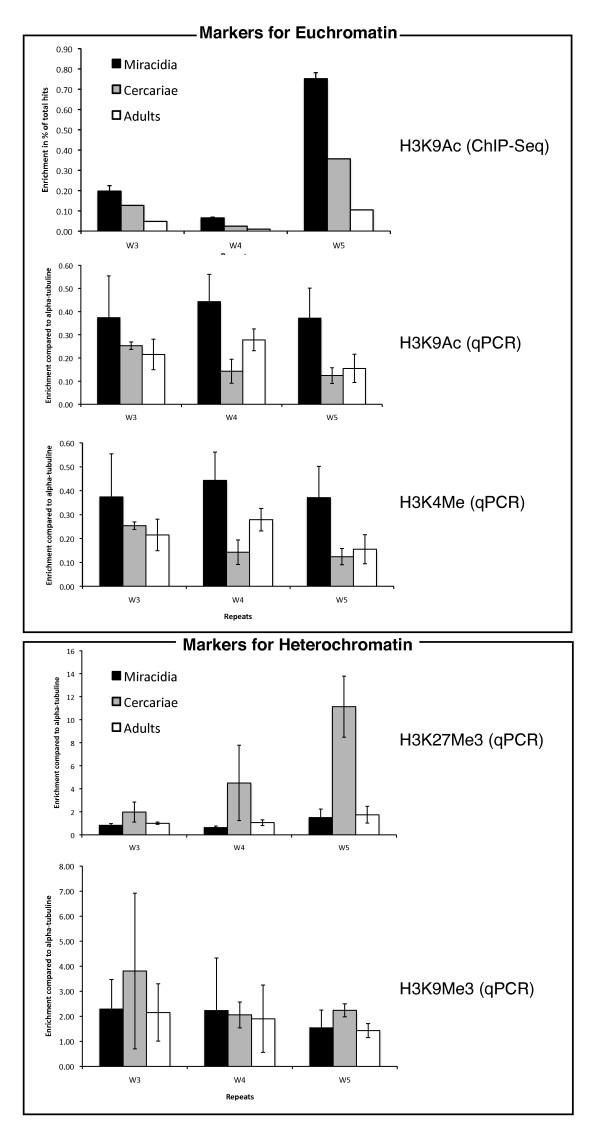
**Chromatin structural changes around female-specific repeats W3, W4 and W5 during the life cycle of *S. mansoni *from miracidia (black) to cercariae (grey) and adult couples (white)**. Measured by ChIP-Seq (upper panel) and ChIP-qPCR (all other panels). Average of three independent ChIP-qPCR experiments, two ChIP-Seq experiments for miracidia and a single ChIP-Seq experiment for cercariae and adults.

Repeat transcription has been linked to chromatin structural changes [30]. We therefore analyzed histone isoforms that could potentially be associated with the female-specific repeats. Chromatin immunoprecipitation followed by massive sequencing (ChIP-Seq) was used to analyze the abundance of acetylated histone H3K9 (H3K9Ac) around the repeats in miracidia, cercariae and adult couples. All 36 female-specific repeats show a characteristic gradual decrease in H3K9 acetylation level from the larval stages to the adult stages. Among the total of 8,594 repeats in the genome, only 1,113 repeats show such a gradual decrease in H3K9 acetylation. The probability that such a pattern could be observed by chance for all 36 W-specific repeats is negligible (the individual term binomial distribution probability is 1.1^-32^). To verify the ChIP-Seq data by ChIP combined with qPCR, we focused on two transcribed repeats (W4 and W5) and one non-transcribed repeat (W3). We used antibodies against H3K9Ac, tri-methylated H3K4 (H3K4Me3) that are characteristic for actively transcribed euchromatin, and the heterochromatin markers tri-methylated H3K9 (H3K9Me3), and tri-methylated H3K27 (H3K27Me3). A region in the body of the alpha-tubulin gene was used as reference for calculating the relative amount of immunoprecipitated DNA. The results are shown in Figure 4. Both euchromatic markers (H3K9Ac and H3K4Me3) are enriched at the repeats in the miracidia stages where transcription was observed. In contrast, there are much fewer euchromatic markers around the repeats in adults. In the qPCR-based experiments, cercariae occupy an intermediate position. Based on the combined ChIP-seq and ChIP-qPCR data, we conclude a clear decrease in H3K9 acetylation from miracidia to cercaria and adults. Also, the abundance of the second euchromatic marker, methylation of H3K4, decreases from miracidia to cercaria and remains constant during the development into adults. The heterochromatic markers H3K9Me3 and H3K27Me3 are abundant in cercaria but low in miracidia and adults. In summary, around the female-specific repeats we observed three distinct types of chromatin structure in the three different life-cycle stages: in miracidia the repeats are clearly euchromatic, in cercaria a large proportion is heterochromatic, and in adults we can find a peculiar chromatin structure without classical euchromatic or heterochromatic markers, but associated with transcriptional silence.

### Histone deacetylase inhibition does not induce transcription of W-specific repeats in adults

We tested whether the observed changes in chromatin structure are a result or the cause of the changes in transcription. If hypoacetylation of histones were the cause of transcriptional inactivation, then inactivation of histone deacetylase would relieve repression. On the other hand, if transcription of repeats is the origin of chromatin structural changes, inhibition treatment should not lead to detectable changes in transcription because each transcriptional increase would reinforce deacetylation and counteract the inhibition. We treated adult parasites with trichostatin A (TSA), an inhibitor of histone deacetylases at increasing concentrations *in vitro*. After 2 hours of treatment with ≥20 µM TSA, mobility changes were observed (worms first straightened up and ceased moving, and convulsive movements were observed at higher concentrations and longer incubation times (Additional file 4)). We then measured the transcription levels for repeats W4, W5 and Sm-alpha-female at 20 µM TSA and for 4 hours. In none of the cases was transcription activated. In contrast, an increase of transcription of retrotransposons Perere3 and Saci7, used as control, was observed (by 45 and 23%, respectively). The lactate dehydrogenase test shows no difference in cytotoxicity between TSA-treated and mock-treated worms.

## Discussion

Despite tremendous advancements in the past, the elements that are responsible for the establishment of sex chromosomes remain still enigmatic. According to Müller's ratchet model, sexual reproduction evolved because deleterious mutations could be eliminated by recombination between the parental autosomes [31]. To maintain isolation of two different sexes, recombination must, however, be repressed (at least partially) between the sex chromosomes. Zones in which recombination is repressed between sex chromosomes were meanwhile identified in many species. Accumulation of repeats on the heterogametic sex chromosome was also found in many examples, although their role is unknown and many authors still consider them as junk DNA. The view that repetitive DNA is non-functional was challenged by the discovery of transcription from repeats on autosomes and the production of small RNA that could be related to heterochromatization events [32]. The presence of large heterochromatic blocks is also a common feature of sex chromosomes. So far, these observations were made in isolation from each other, and generally in different species, which makes the construction of a hypothetical model difficult. Here we present for the first time a comprehensive analysis of sequence composition, gene and repeat content, chromatin structure and repeat transcription of the sex-specific chromosome regions of the Z and W chromosomes of our biological model *S. mansoni*. Recombination repression has been described before in this region of interest [22]. Our data, in relation to previous reports, allows the current models for the suite of events that led to sex chromosome differentiation in *S. mansoni *to be refined and could represent a general model for this process in species with genetic sex determination of the Z/W type.

### Z- and W-specific sequences

Criscione *et al*. [22] identified a region of 20 scaffolds in which recombination repression was observed and suggested that these are Z-specific sequences. We indeed found a male/female sequence reads hit and/or qPCR ratio of ≥1.5 for 13 of these scaffolds, indicating an overrepresentation in the male genome. However, seven scaffolds in this region showed no disequilibrium of hit counts and/or qPCR between males and females (male/female hit ratio ≤1.4), that is, the sequences are not specific to the Z chromosome (Figure 1). In other words, recombination is repressed but the homologous sequences on the sister chromosomes are still present. We find at least two blocks of sequences that are shared between the Z and W chromosome located in the large region with recombination repression. This result was confirmed with the most recent version of the genome assembly. We see three possible conclusions that can be drawn from our results. Either the Z/W sequence blocks are inverted, and additionally or alternatively the sequences are heterochromatic, thus preventing recombination. It is also possible that the scaffolds in the original assembly of the *S. mansoni *genome were chimeric. Indeed, of the 48 scaffolds originally found in linkage group Z/W [22], 4 are on other chromosomes in the 5.2 assembly. It will be difficult to formally exclude the possibility that our results are due to misassembly.

We did not find any paralogues to sex determination genes among the predicted genes on the Z-specific scaffolds. The specific region of the W chromosome is largely composed of large satellite blocks of at least 36 different W-specific repeats. These repeats are abundant on the W chromosome but our PCR analysis on different male individuals indicates that these sequences can also sometimes be found on other chromosomes. The strength of the PCR signal suggests, however, that they are present in very low copy number there. Analysis of the genomic sequence shows that they can occur intermingled with other repeats on autosomal scaffolds as individual sequences or as small blocks of up to five repeats in tandem. Our understanding of these results is that these repeats exist as large satellite blocks on the W chromosome but can occasionally be transferred to autosomes by a so far unknown mechanism. Such a behavior was described for W1 [28] and could depend on the chromatin structure around the repeats and/or flanking regions. Several of these W-specific repeats are transcribed in the miracidia and cercariae stages but never in the adults.

### Role of W-specific repeats

**Figure 5:**
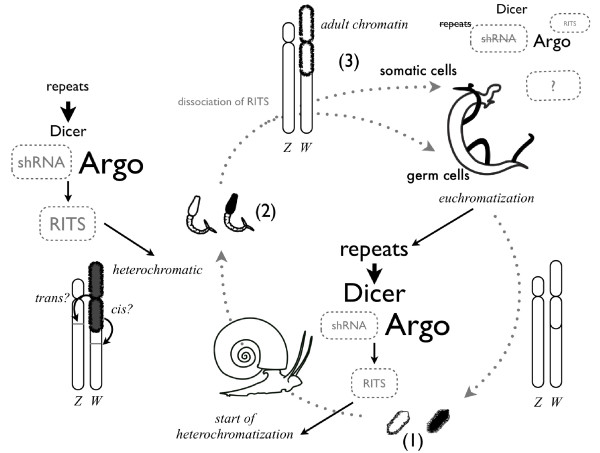
**Hypothetical model of the relationship between W-specific repeat transcription and heterochromatin formation**. In black, experimentally confirmed situation; in grey, hypothetical elements. Letter size corresponds to relative strength of the phenomenon. (1) Miracidia do not show sex dimorphism. The W chromosome is euchromatic and repeats are transcribed. Large amounts of Dicer and Argonaute proteins are present [39,40,58]. Dicer could produce small heterochromatic RNA (shRNA) that could bind to Argonauts and could build a RITS (RNA-induced initiation of transcriptional gene silencing) complex similar to those in yeast [42] that initiates heterochromatization around the repeat region of the W chromosome. (2) After infection of the snail host, cercariae are produced. Also, these larvae do not show sex dimorphism. Repeats are still transcribed but transcription of Dicer has decreased [39]. Accumulation of RITS complex around the repeat region progressively leads the loss of its euchromatic character. Finally (3), in dimorphic adults, the repeat region becomes transcriptionally inactive, and heterochromatin is maybe locked by HP1 or related chromatin proteins such as KDM2A [59], or a second histone modification mark is missing [60]. Dicer and Argonaut are less abundant. RITS is no longer necessary. Sexual reproduction occurs during this stage. Recombination is repressed by non-permissive chromatin. Late in germ cell production or during embryogenesis, erasure of chromatin marks occurs (epigenetic reset). The cycle restarts with a euchromatic W chromosome.

In most species that possess sex chromosomes of the Y or W type it was found that (i) repetitive sequences accumulate on these chromosomes, (ii) large regions are heterochromatic and (iii) these chromosomes deteriorate or are completely absent in the extreme case. We show that the W chromosome in *S. mansoni *is no exception to this rule. What is unknown, however, is the suite of events in the evolution of sex chromosomes and the role of the different elements in sex determination. We believe our present study sheds some light on this matter. Heterochromatization of the W chromosome in schistosomes has been known for a long time and has been even used as a marker for sex identification in morphologically indistinguishable cercariae [14,15,33]. Based on cytogenetic analysis, some authors argued that heterochromatization of the W starts in miracidia [14]. Since it is impossible to determine chromosome banding in miracidia and then reuse the larvae for infection and production of adults, these results are difficult to verify. Our results clearly show that the repeats that are located in the W heterochromatic region carry a euchromatic signature in miracidia and lose their euchromatic character progressively during the development into adults. This process is accompanied by a decrease of transcription until complete silencing of the repeats in the sexually mature adult stage. During the miracidia to cercaria transition - that is, precisely when sexual dimorphism starts to develop - the repeats heterochromatize. Sex-specific repeats are found in many species [34-36]. In some cases transcription has been described and it was suspected that these repeats play a role in the sex determination process [37,38]. The transcription of repetitive elements of the satellite type in *S. mansoni *is particularly interesting in the light of the recent discovery of stage-dependent expression of the elements that constitute the RNA interference (RNAi) pathway in schistosomes [39,40]. In many organisms RNAi and chromatin structural changes are linked [32,41-43] and it is tempting to speculate that transcription of W-specific repeats is actually the origin of chromatin compaction on the W chromosome during the life cycle. A hypothetical scheme is shown in *Figure *5. In our model, reset of the repeat chromatin structure occurs during early embryogenesis (formation of miracidia). In the miracidia, repeats are euchromatic and several of them are transcribed. Transcripts are processed through a pathway that has similarity to RNAi and a hypothetical repeat-induced silencing complex is formed that induces the formation of heterochromatin around the repeats. At this stage, miracidia have infected the mollusk host and develop via sporocyst stages into cercaria. In cercaria, most of the repeats are heterochromatic and not transcribed. We hypothesize that the heterochromatization extends beyond the repeat frontiers and that nearby loci are silenced. If a sex determination locus is found among these loci, the heterochromatization would lead to a dose effect that could be the origin of the formation of the female adult phenotype. Once the task of silencing this locus in *cis *(or *trans*) is accomplished, repeats are not anymore transcribed and the chromatin structure of the pericentromeric W chromosome is fixed into an unknown but transcriptionally silent configuration. We can only speculate about the proteins that are involved since our data indicate that neither the euchromatic markers H3K9Ac and H3K4Me3 nor the heterochromatic markers H3K9Me3 and H3K27Me3 are abundant. This model is supported by our finding that *in vitro *treatment of adults does not lead to detectable transcription from the W-specific repeats while autosomal retrotransposons can be activated.

One could argue that the function of repeat-induced silencing is purely defensive and down-regulates retrotransposon expression in general. Such a mechanism was described as the repeat-associated small interfering RNA (rasiRNA)-mediated pathway [44] in *Drosophila *ovary cells and is believed to protect the (female) germ line from transposable elements. If this were the case for *S. mansoni*, transcription should be observed in the ovary. Our data do not support this view.

## Conclusions

Most authors agree that suppression of recombination is an initial event in sex chromosome emergence, although it is not clear by what mechanism it is caused. Chromosome rearrangements (for example, inversions) or the action of modifier genes have been proposed (reviewed, for example, in [45]). Other authors see conformation differences (chromatin structural changes, differences in replication timing) as the origin for recombination inhibition [3,5]. Accumulation of repeats is a general feature of Y/W-type chromosomes. Some consider it an important feature with unknown function [36], while others see repeat accumulation as the result of recombination suppression [1] or solely as a genome defense mechanism [7], placing it late in the suite of events that characterize evolution of sex chromosomes.

With the present work we contribute two new elements that allow us to exclude some of the current hypotheses and to refine others. First, we show that the presence of satellite repeats on the W chromosome does not lead in all life cycle stages to heterochromatization. Consequently, it is not their presence itself that induces the heterochromatin formation. We show that all W-specific repeats are euchromatic in the miracida stage. Our ChIP-Seq data tell us that this is not a general feature of autosomal and pseudoautosomal repeats, but specific for the W-specific satellites. Second, we demonstrate that the euchromatization occurs concomitantly with transcription and that transcription always precedes heterochromatization.

Based on these findings, we propose two not necessarily exclusive scenarios for the emergence of sex chromosomes. In the first model, transcription of non-coding RNA from repetitive DNA elements was the initial event in sex chromosome evolution of schistosomes. Non-coding RNA would have induced heterochromatization and suppression of recombination. Both favored expansion of repeats and organization in large blocks (satellites). Satellite expansion would have reinforced the system and led finally to the beginning of genetic changes in the W chromosome. The very basal phylogenetic position of leuphotrochozoans such as *S. mansoni *permits a general model for the main stages of sex chromosome evolution to be proposed: the establishment of a sex-determining region, recruitment of repeats for production of non-coding RNA, RNA-directed heterochromatization and repeat expansion, local suppression of recombination, and shrinkage of the chromosome by deletion.

In the second model, a small mutation and/or local heterochromatization could have been the initial event, leading to recombination repression in the first place. Repetitive DNA accumulated subsequently. During germ cell formation or during early embryogenesis euchromatization occurs. Cytogenetic evidence in other species in which the female is the heterogametic sex shows that the W chromosome is often condensed in somatic cells, and becomes euchromatic in early oocytes (reviewed in [46]). This releases transcription repression and repeats are transcribed, leading subsequently to heterochromatization. Our preliminary data suggest that chromatin structural changes do not occur in *trans *- that is, not on the Z chromosome but on the adjacent regions of the W chromosome (not shown).

We cannot formally exclude that sex determination is based on a specific protein-coding gene that is absent or present on the W chromosome. But we show that the most pronounced difference in transcription between ZZ and ZW individuals is at the level of 'non-coding' RNA. We therefore favor the hypothesis that sex differentiation in *S. mansoni *is based on developmental stage-dependent tagging of the W chromosome by non-coding RNA and a chromatin marking system. Our model predicts that chromatin structural changes influence transcription of one or several genes in the close vicinity of the core heterochromatic region and that transcriptional activation or inactivation of these leads to morphological and/or physiological changes that are the bases for development of the male and female phenotypes in the adult stage.

## Materials and methods

### Parasite culture and drug treatment

Eggs were axenically recovered from 60-day infected hamster livers and miracidia were hatched from eggs in 5 ml of spring water over 2 to 3 hours under light. Miracidia were concentrated by sedimentation on ice for 15 minutes. Cercariae were recovered from infected snails (4 weeks post-infection) and collected by pipetting. They were then concentrated by cold centrifugation (4°C) at 1,200 rpm for 5 minutes and the supernatant was removed. Eight-week-old adult worms were recovered by portal perfusion of hamsters with 0.8% (w/v) NaCl and 0.8% (w/v) trisodium citrate [47]. If necessary, miracidia, cercariae and adults were kept at -80°C.

For infection with a single sex, *B. glabrata *snails 4 to 5 mm in diameter were individually exposed to a single miracidium in 5 ml of springwater. The snails were then each isolated and maintained in round, clear plastic containers for 24 hours and kept all together for 5 weeks. Snails were fed fresh lettuce *ad libitum *and the water was maintained at 25°C and changed weekly. The photoperiod during the entire experiment was equilibrated to 12 hours light:12 hours dark [48].

Adults were recovered by portal perfusion of hamsters. Ten individuals were kept in 250 µl RPMI medium (Invitrogen-Gibco, Carlsbad, USA) and treated with an ethanol solution of the histone deacetylase inhibitor TSA (Invitrogen) at different final concentrations (2 µM, 20 µM, 50 µM, 100 µM and 200 µM). To the untreated control, a corresponding volume of ethanol was added. The cytotoxic effect of the drug was measured using the Roche Cytotoxicity Detection Kit (Roche no. 04744926001), which is based on the measurement of lactate dehydrogenase activity released from dead and lysed cells into the supernatant [49]. Behavior was observed every hour until 6.5 hours and after 21 hours of treatment. Individuals were filmed with a conventional numerical camera adapted to a stereomicroscope after 5, 6.5 and 21 hours of treatment.

### Sequencing of genomic DNA, alignment, and assembly of repeats

Solexa sequencing was performed at the sequencing facilities of GenomiX Montpellier (France) on a Genome Analyzer II (Illumina) by single end sequencing (36 bp) according to the manufacturer's protocol. The software SOAP is usually employed to map unique sequences and reject repetitive sequences. We took advantage of this algorithm and used SOAP 2.17 [50], evoking the -u and -r 0 options to split the sequence reads into those corresponding to unique or repetitive sequences. The resulting fasta files of unmapped reads (-u) was assembled with velvet using a coverage cutoff of 4 and a minimum contig length of 80 bp. For a second assembly round Sequencher v4.5 was used with minimum match 93%, minimum overlap 60 bp.

### *In silico *analysis

Velvet-assembled repeats were then used for the whole-genome *in silico *subtractive hybridization (WISH) procedure [24]. This method compares different massive sequencing datasets with a reference genome and identifies sequences that are under-represented in one data set. Censor [51], Teclass [52] and blast [53] were used for repeat annotation.

For identification of genes in the vicinity of W-specific repeats, all repeat sequences were compared to the genome using blast searches of the SchistoDB database [54] and genes 5 kb upstream and downstream of regions containing these repeats were manually analyzed.

### Confirmation of sex-specific sequences by PCR

PCRs were carried out in a final volume of 25 µl containing 0.2 µmol of each oligonucleotide primer (Additional file 5), 0.2 mmol of each dNTP (Promega), 0.625 U of GoTaq polymerase (Promega) used with the recommended buffer and completed to the final volume with DNase-free water. The PCR program consisted of an initial denaturation phase at 95°C for 5 minutes followed by 20 cycles at 95°C for 30 s, 60°C for 90 s, 72°C for 30 s and a final extension at 72°C for 5 minutes. The PCR products were separated by electrophoresis through a 2% TBE agarose gel.

### FISH on *S. mansoni *metaphases

Metaphase spreads were prepared essentially as described by Hirai and LoVerde [55]. Sporocysts were obtained by dissection of two to three snails, each infected with five miracidia, at 28 to 29 days post-infection. Probes for repetitive DNA were prepared by cloning PCR products (for primers see Additional file 5) on genomic DNA as template into pCR2.1-TOPO (Invitrogen #K4510-20). Clones were sequenced to confirm the repeat assembly, labeled with the BioPrime DNA labeling system (Invitrogen #18094-011) and hybridized as described before [55]. Chromosomes were counterstained with propidium iodide and observed under an epifluorescence microscope (AKIOSKOP 2, Zeiss) equipped with a Leica DC 300 FX digital camera. Between 7 and 34 female metaphases were studied for each repeat.

### RNA extraction, cDNA synthesis and qPCR

Total RNA was purified from three independent preparations of larvae and adults. For the larval stages, RNA was extracted from 10,000 miracidia and 10,000 cercariae using 500 µl Trizol (Invitrogen). Fifty adult couples were solubilized in 500 μl Trizol with a MagNA Lyser and Green beads (Roche). RNA was treated with DNase I (Invitrogen) for 15 minutes at 37°C, followed by inhibition of the enzyme for 10 minutes at 65°C. PCR of 28s rDNA was used to test for genomic DNA contaminations. The DNase I treatment was repeated as many times as necessary to eliminate contaminations with genomic DNA. RNA was purified with the QIAGEN RNeasy kit. First strand cDNA was synthesized using 10 μl of the total RNA preparation, in a final volume of 20 μl (10 mM dNTPs, 0.1 M DTT, 40 U RNase out, 0.15 μM random primers) with 200 U of SuperScript II RT (Invitrogen). After reverse transcription, the cDNAs were purified with the PCR clean-up system (Promega) and eluted into 40 μl 10 mM Tris/Cl (ph 7.5). Real-time PCR analyses were performed using the LightCycler 2.0 system (Roche Applied Science) and LightCycler Fast-start DNA Master SYBR Green I kit (Roche Applied Science).

qPCR amplification was done with 2.5 μl of cDNA in a final volume of 10 μl (3 mM MgCl_2_, 0.5 μM of each primer, 1 μl of master mix). Primers were designed with the LightCycler Probe design software or the primer3plus web based interface [56]. The following protocol was used: denaturation, 95°C 10 minutes; amplification and quantification (40 cycles), 95°C for 10 s, 60°C for 5 s, 72°C for 16 s; melting curve, 60 to 95°C with a heating rate of 0.1 C/s and continuous fluorescence measurement, and a cooling step to 40°C. For each reaction, the crossing point (Ct) was determined using the 'fit point method' of the LightCycler Software 3.3. PCR reactions were done in duplicates and the mean value of Ct was calculated. 28s rRNA was used as an internal control and the amplification of a unique band was verified by electrophoresis through 2% TBE agarose gels for each qPCR product. Primer sequences and expected PCR product size are listed in Additional file 5. For all qPCR, efficiency was at least 1.89.

### Chromatin status analysis by ChIP and qPCR

Native ChIP and ChIP-Seq were performed as described before [57]. In brief, antibodies against histone isoforms (Table 3) were used to precipitate chromatin in sporocysts, cercaria and adults. The resulting DNA was analyzed either by ChIP-Seq or qPCR. ChIP-Seq data are available at the NCBI SRA under accessions SRX088545, SRX088544, SRX088543 and SRX087825. For ChIP-Seq analysis, a repeat pseudogenome was constructed in which each identified repeat sequence occurred only once. Then SOAP2 [50] was used to align roughly 100,000 36-bp reads for miracidia of two strains (GH2 and BRE), cercaria and adult couples (both GH2) to this pseudogenome. Hit counts for each repeat were normalized by the total number of aligned reads and compared for the different stages.

**Table 3:** Antibodies used for native ChIP (N-ChIP)

Antibody	Host	Product	Lot	Saturating quantity used for N-ChIP^a^
H3K9ac	Rabbit	Upstate, 07-352	DAM16924924	8 µl
H3K4me3	Rabbit	Upstate, 04-745	NG1680351	4 µl
H3K9me3	Rabbit	Abcam, Ab8898	733951	4 µl
H3K27me3	Rabbit	Diagenode, pAb-069-050	A29900242	8 µl

^a^Saturating quantities for H3K9ac and H3K9me3 antibodies were previously determined [56]. Saturating quantities for H3K4me3 and H3K27me3 were determined in this study by a titration experiment (data not shown).

## Abbreviations

bp: base pair; ChIP: chromatin immunoprecipitation; ChIP-qPCR: chromatin immunoprecipitation followed by quantitative PCR; ChIP-Seq: chromatin immunoprecipitation followed by massively parallel sequencing; EST: expressed sequence tag; FISH: fluorescence *in situ *hybridization; H3K27Me3: histone H3 tri-methylated on lysine 27; H3K4Me3: histone H3 tri-methylated on lysine 4; H3K9: histone H3 lysine 9; H3K9Ac: histone H3 acetylated on lysine 9; H3K9Me3: histone H3 tri-methylated on lysine 9; NCBI SRA: Sequence Read Archive at the National Center for Biotechnology Information; PCR: polymerase chain reaction; qPCR: quantitative PCR; RNAi: RNA interference; TSA: trichostatin A.

## Competing interests

The authors declare that they have no competing interests.

## Authors' contributions

JMJL did most of the experimental work and wrote the manuscript, CC conducted ChIP experiments, JB performed TSA treatment and edited the manuscript, MF performed ChIP-Seq, JP and DC did PCR and qPCR confirmation of W- and Z-specific sequences, CP contributed to data analysis, AZN did part of the bioinformatics work, and CG designed the experiment, performed FISH experiments, edited the manuscript and analyzed the massive sequencing data.

## Supplementary Material

Additional file 1 **List of male-specific scaffolds with putative genes**. Click here for file

Additional file 2 **Video of adult schistosomes treated with TSA at 100 µM**. Individuals were filmed with a conventional numerical camera adapted to a stereomicroscope after 5 hours of treatment. Click here for file

Additional file 3 **Video of adult mock-treated schistosomes**. Individuals were filmed with a conventional numerical camera adapted to a stereomicroscope after 5 hours of treatment. Click here for file

Additional file 4 **Photographs of ethidium bromide stained PCR products after migration through 2% agarose gels**. PCR amplification was used to confirm size and sex-specificity of assembled W-specific repeats. Genomic DNA of two female (F1, F2) and two male individuals (M1, M2) was used as template. Click here for file

Additional file 5 **Primers used in this study**. Click here for file
