# Supplementary material for: Chromatin structural changes around satellite repeats on the female sex chromosome in Schistosoma mansoni and their possible role in sex chromosome emergence
Source: Genome Biol. 2012 Feb 29;13(2):R14. doi: 10.1186/gb-2012-13-2-r14 (PMC3701142; doi:10.1186/gb-2012-13-2-r14)
Supplement: Additional file 1 — List of male-specific scaffolds with putative genes. [file gb-2012-13-2-r14-S1.DOC]

**Supplementary Table 1: Z-specific scaffolds with putative genes**

| **scaffold overrepresented in male based on eland hits** | **Genes** | **SchistoDB function** | **similarity to… / comments** |
| --- | --- | --- | --- |
| Smp_scaff000398 | Smp_169540 | heterogeneous nuclear ribonucleoprotein-related, putative |  |
|  | Smp_169550 | amine oxidase, putative |  |
|  | Smp_169560 | lis1-interacting protein nude, putative |  |
|  | Smp_169570 | glycerol-3-phosphate dehydrogenase, putative |  |
|  | Smp_169580.1 |  |  |
|  | Smp_169580.2 |  |  |
| Smp_scaff018906 |  |  |  |
| Smp_scaff000301 | Smp_080370.1 |  |  |
|  | Smp_080370.2 |  |  |
|  | Smp_165430 |  |  |
|  | Smp_165440 | netrin receptor unc5, putative |  |
|  | Smp_165450 | *many paralogs* |  |
|  | Smp_080420 | transcription factor AP-1/JUN, putative |  |
|  | Smp_165470 | epidermal growth factor receptor, putative |  |
| Smp_scaff001995 | Smp_104710 |  |  |
|  | Smp_104720 | NAD dependent epimerase/dehydratase, putative |  |
|  | Smp_104730 | DNAj homolog subfamily B member 4,, putative | **spermatogenesis apoptosis-related protein [Xenopus (Silurana) tropicalis]** |
|  | Smp_180650 |  |  |
|  | Smp_180660 |  |  |
|  | Smp_180670 |  |  |
|  | Smp_104760 |  |  |
|  | Smp_180680 | similar to armadillo repeat containing 2 isoform 2 |  |
|  | Smp_104790 | leucine rich repeat-containing, putative |  |
|  | Smp_180690 | tiptop, putative |  |
| Smp_scaff000218 | Smp_067500 |  |  |
|  | Smp_067510 | similar to Clusterin associated protein 1 |  |
|  | Smp_067520 | jun-related protein, putative |  |
|  | Smp_158720 | scy1(yeast) protein kinase-like |  |
|  | Smp_067540 | similar to Vacuolar protein sorting-associated protein 28 homolog |  |
|  | Smp_158730 |  | **sperm flagellar protein 2 KPL2** |
|  | Smp_158740 |  |  |
|  | Smp_067580 |  |  |
|  | Smp_067590.1 |  |  |
|  | Smp_067590.2 |  |  |
|  | Smp_158750 | forkhead protein/ forkhead protein domain, putative |  |
|  | Smp_158760 | dynein heavy chain, putative |  |
|  | Smp_067620 | dynein heavy chain, putative |  |
|  | Smp_158770 |  |  |
|  | Smp_158780 | adapter-related protein complex 1 gamma subunit (gamma-adaptin) |  |
|  | Smp_158790 |  |  |
|  | Smp_158800 |  |  |
| Smp_scaff000465 | Smp_092530.1 | adapter-related protein complex 1 gamma subunit (gamma-adaptin) |  |
|  | Smp_092530.2 | adapter-related protein complex 1 gamma subunit (gamma-adaptin) |  |
| Smp_scaff000514 | Smp_173080 |  |  |
|  | Smp_093100 | U2 snrnp auxiliary factor, small subunit, putative |  |
|  | Smp_093110 |  |  |
|  | Smp_173090 | eyes absent homolog, putative |  |
| Smp_scaff000425 | Smp_171590 | dual specificity protein phosphatase cdc14, putative |  |
|  | Smp_171600 |  |  |
|  | Smp_171610 | dual specificty protein kinase-ttk, putative |  |
|  | Smp_171620 | methylthioadenosine phosphorylase, putative |  |
|  | Smp_090520 | purine nucleoside phosphorylase, putative |  |
|  | Smp_171630 | sodium-dependent phosphate transporter, putative |  |
|  | Smp_171640 |  |  |
|  | Smp_171650 | EPIDERMAL GROWTH FACTOR RECEPTOR SUBSTRATE 15 related |  |
|  | Smp_171660 |  |  |
|  | Smp_171670 | wd-repeat protein, putative |  |
|  | Smp_171680 |  |  |
|  | Smp_171690 |  |  |
|  | Smp_171700 | **myst histone acetyltransferase, putative** |  |
|  | Smp_171710 | **structural maintenance of chromosomes smc2, putative** |  |
| Smp_scaff001883 | Smp_179190 |  |  |
| Smp_scaff001948 | none |  |  |
| Smp_scaff000059 | Smp_137430 | spliceosomal protein sap, putative |  |
|  | Smp_028190 | methylthioadenosine phosphorylase, putative |  |
|  | Smp_028200.1 | Troponin I (TnI), putative | expressed in male |
|  | Smp_028210 | calcyphosine/tpp, putative |  |
|  | Smp_137440 |  |  |
|  | Smp_028230 |  |  |
|  | Smp_137450 |  |  |
|  | Smp_137460 | cytoplasmic polyadenylation element binding protein (cpeb), putative |  |
|  | Smp_137470 | phospholipase C beta, putative |  |
|  | Smp_137480 |  |  |
|  | Smp_137490 |  |  |
|  | Smp_028330 |  |  |
|  | Smp_028340 | brix domain containing protein, putative |  |
|  | Smp_195010 | hydroxymethylglutaryl-CoA synthase, putative |  |
|  | Smp_137500 |  |  |
|  | Smp_137510 |  |  |
|  | Smp_028360.2 | tubulin epsilon chain, putative |  |
|  | Smp_028370 |  |  |
|  | Smp_137520 |  |  |
|  | Smp_028390 |  |  |
|  | Smp_137530 | stomatin-related | expressed in male |
|  | Smp_028410 |  |  |
|  | Smp_137540 | disulfide oxidoreductase, putative |  |
|  | Smp_028430 | ring finger, putative |  |
|  | Smp_028440.1 | adenosylhomocysteinase, putative |  |
|  | Smp_028440.2 | adenosylhomocysteinase, putative |  |
|  | Smp_028440.3 | adenosylhomocysteinase, putative |  |
|  | Smp_028450 |  |  |
|  | Smp_137550 | dynein heavy chain, putative |  |
|  | Smp_137560 |  |  |
|  | Smp_028480 |  |  |
|  | Smp_028490 | 1-acylglycerol-3-phosphate acyltransferase, putative |  |
|  | Smp_028500 | **caspase related** | expressed in male |
|  | Smp_137570 | helicase, putative |  |
|  | Smp_137580 | helicase, putative |  |
|  | Smp_028550 | helicase, putative |  |
|  | Smp_137590 | ribosomal rna methyltransferase, putative |  |
|  | Smp_137600 |  |  |
|  | Smp_137610 | focal adhesion kinase, putative |  |
|  | Smp_028620 | exocyst complex component sec6, putative |  |
|  | Smp_028630 | exocyst complex component sec6, putative |  |
|  | Smp_028640 |  |  |
|  | Smp_028650 |  |  |
|  | Smp_137620 | Uveal autoantigen with coiled-coil domains and ankyrin repeats protein, putative |  |
|  | Smp_028670.1 | carbonic anhydrase II (carbonate dehydratase II), putative | drug target |
|  | Smp_028670.2 | carbonic anhydrase II (carbonate dehydratase II), putative |  |
|  | Smp_028690 |  |  |
|  | Smp_137630 |  |  |
| Smp_scaff000044 | Smp_022560.1 | 60S ribosomal protein L21, putative |  |
|  | Smp_022560.2 |  |  |
|  | Smp_134240 |  |  |
|  | Smp_022570.2 | ATXN7L3-like protein |  |
|  | Smp_134250 | heparan sulfate n-deacetylase/n- sulfotransferase, putative |  |
|  | Smp_134260 | **MAPk, putative** |  |
|  | Smp_022610 | musculin, putative |  |
|  | Smp_134270 |  |  |
|  | Smp_022620 |  |  |
|  | Smp_022630 |  | **similar to testis expressed gene 2 [Rattus norvegicus]** |
|  | Smp_022640 | 60S ribosomal protein L13 (BBC1 protein homolog), putative |  |
|  | Smp_134280 |  |  |
|  | Smp_022660 | wd-repeat protein, putative |  |
|  | Smp_134290 | serine-rich repeat protein , putative |  |
|  | Smp_134300 | beta-hexosaminidase B, putative |  |
|  | Smp_134310 | brg-1 associated factor, putative |  |
|  | Smp_022700.1 | protein tyrosine phosphatase-like protein |  |
|  | Smp_022710 | kif1, putative |  |
|  | Smp_022730 | nucleolar protein family A member 1 (snornp protein gar1), putative |  |
|  | Smp_022740 | tumor necrosis factor induced protein, putative |  |
|  | Smp_134330 | kif1, putative |  |
|  | Smp_022760 |  |  |
|  | Smp_134340 |  |  |
|  | Smp_134350 | **thyrotropin-releasing hormone receptor 1,, putative** |  |
|  | Smp_134360 |  |  |
|  | Smp_134370 | rna 3' terminal phosphate cyclase, putative |  |
|  | Smp_134380 |  |  |
|  | Smp_022810.1 | **rab-2,4,14, putative** |  |
|  | Smp_134390 | sterol o-acyltransferase, putative |  |
|  | Smp_134400 | arp2/3 complex subunit 41-related |  |
|  | Smp_134410.1 | monocarboxylate transporter, putative |  |
|  | Smp_022850.2 |  |  |
|  | Smp_134420 | mRNA turnover protein 4 mrt4, putative |  |
|  | Smp_022870 |  | expressed in male |
|  | Smp_022890 |  |  |
|  | Smp_134430 | tolloid-related |  |
|  | Smp_134440.1 | malic enzyme, putative |  |
|  | Smp_134450 |  |  |
|  | Smp_134460 | ancient conserved domain protein 2 (cyclin m2), putative |  |
|  | Smp_scaff000576 |  |  |
|  | Smp_174270 |  |  |
|  | Smp_174280 | dynein heavy chain, putative |  |
|  | Smp_174290 |  |  |
|  | Smp_174300 |  |  |
| Smp_scaff000019 | Smp_128310.1 | xylosyltransferase, putative |  |
|  | Smp_128310.2 | xylosyltransferase, putative |  |
|  | Smp_128320 |  |  |
|  | Smp_128330 | ets-related |  |
|  | Smp_128340 | NAD dependent epimerase/dehydratase, putative |  |
|  | Smp_128350 |  |  |
|  | Smp_128360 | malic enzyme, putative |  |
|  | Smp_128370 | glutamate synthase, putative |  |
|  | Smp_128380.3 | glutamate synthase, putative |  |
|  | Smp_128380.2 | glutamate synthase, putative |  |
|  | Smp_128380.1 | glutamate synthase, putative |  |
|  | Smp_011560 | tetraspanin, putative |  |
|  | Smp_011570.1 | 40S ribosomal protein S4, putative |  |
|  | Smp_011570.2 | 40S ribosomal protein S4, putative |  |
|  | Smp_011590.1 | prolyl endopeptidase (prolyl oligopeptidase), putative |  |
|  | Smp_011590.2 | prolyl endopeptidase (prolyl oligopeptidase), putative |  |
|  | Smp_011600 |  |  |
|  | Smp_011610 |  |  |
|  | Smp_128390 |  |  |
|  | Smp_128400.1 |  |  |
|  | Smp_128400.2 |  |  |
|  | Smp_128410.3 |  |  |
|  | Smp_011640.1 |  |  |
|  | Smp_011640.2 |  |  |
|  | Smp_011660.2 | calcium/calmodulin dependent protein kinase II, putative |  |
|  | Smp_011660.1 | calcium/calmodulin dependent protein kinase II, putative |  |
|  | Smp_011660.3 | calcium/calmodulin dependent protein kinase II, putative |  |
|  | Smp_011680.3 | CD36-like class B scavenger receptor |  |
|  | Smp_011680.1 | CD36-like class B scavenger receptor |  |
|  | Smp_011690 |  |  |
|  | Smp_011700 | tyrosine protein kinase, putative |  |
|  | Smp_011710 |  |  |
|  | Smp_011720 |  |  |
|  | Smp_128420 |  |  |
|  | Smp_011740 | eppb9, putative |  |
|  | Smp_011750 |  |  |
|  | Smp_128430 | cd36 antigen, putative |  |
|  | Smp_128440 | run and fyve domain containing protein, putative |  |
|  | Smp_128450 |  |  |
|  | Smp_128460 | type V p-type atpase isoform, putative |  |
|  | Smp_128470 |  |  |
|  | Smp_011830 |  |  |
|  | Smp_128480 | protein kinase C, putative |  |
|  | Smp_011860 | transcription factor LCR-F1 , putative |  |
|  | Smp_011870 |  |  |
|  | Smp_128490 | tomosyn, putative |  |
|  | Smp_128500 |  |  |
|  | Smp_128510 |  |  |
|  | Smp_128520 |  |  |
|  | Smp_128530 |  |  |
| Smp_scaff018900 | Smp_193810 |  |  |
|  | Smp_120380 | **testis specific leucine rich repeat protein, putative** |  |
