# Supplementary figures and images for: Chromatin structural changes around satellite repeats on the female sex chromosome in Schistosoma mansoni and their possible role in sex chromosome emergence
Source: Genome Biol. 2012 Feb 29;13(2):R14. doi: 10.1186/gb-2012-13-2-r14 (PMC3701142; doi:10.1186/gb-2012-13-2-r14)

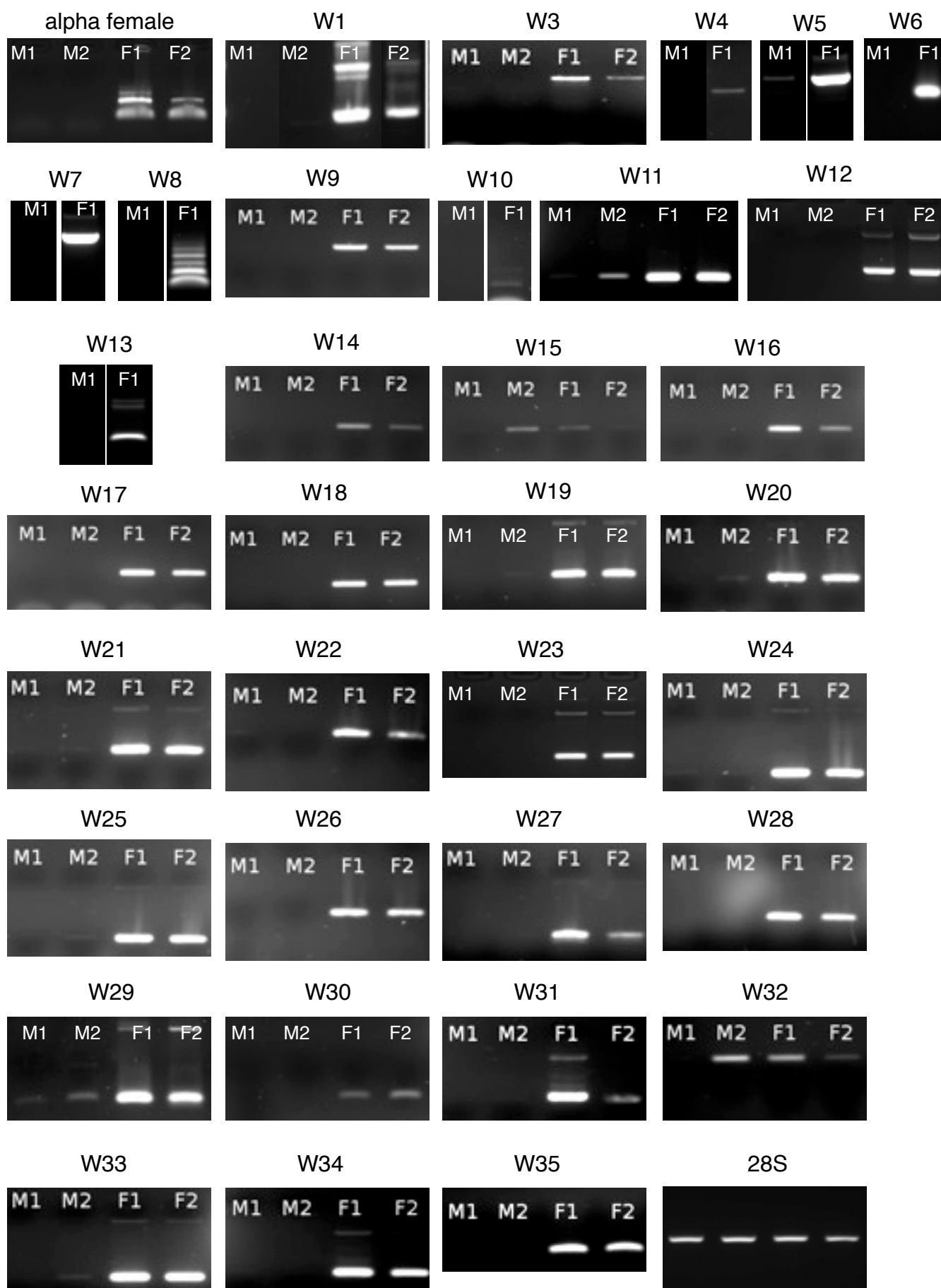

Supplement: Additional file 4 — Photographs of ethidium bromide stained PCR products after migration through 2% agarose gels. PCR amplification was used to confirm size and sex-specificity of assembled W-specific repeats. Genomic DNA of two female (F1, F2) and two male individuals (M1, M2) was used as template. [file gb-2012-13-2-r14-S4.PDF]
