# Supplementary material for: Chromatin structural changes around satellite repeats on the female sex chromosome in Schistosoma mansoni and their possible role in sex chromosome emergence
Source: Genome Biol. 2012 Feb 29;13(2):R14. doi: 10.1186/gb-2012-13-2-r14 (PMC3701142; doi:10.1186/gb-2012-13-2-r14)
Supplement: Additional file 5 — Primers used in this study. [file gb-2012-13-2-r14-S5.DOC]

**Supplementary Table 2: Primers used in this study**

| **Targets** | | **Forward primer** | **Reverse primer** | **Expected size (bp)** | **Annealing temperature** |
| --- | --- | --- | --- | --- | --- |
| PCR for confirmation of female-specific repeats | | | | | |
| SMAlphafem-1 | | tgcacaagtgagtggcctgtggg | tggatgtacctgcatcccgtgt | 120 | 60°C |
| W3 | | tctactgctgattggttgcg | tgcacaatcacagctccttc | 610 | 60°C |
| W4 | | gtgaccgtgttttggtgttg | gttatgagggttgaccacgc | 176 | 60°C |
| W5 | | caagctcgcgtacgatgata | gcttcacgtgagtggtgaga | 295 | 60°C |
| W9 | | tcgtcaaacacctcatccaa | gctggcgaagttttcaatgt | 208 | 60°C |
| W11 | | ctacgaaacgcctgtcttcc | tgcatcatggtggtttcatt | 205 | 60°C |
| W14 | | tgaattggacttcgcaaatg | atgttttcaagtgaacaaatacgg | 150 | 60°C |
| W16 | | aatctctattgtgaagatcaaatcaa | tgaacaacctgaaaagacgttg | 151 | 60°C |
| W17 | | cgatctgtactgttcacagactacc | ctcagttcattctgcactgtca | 155 | 60°C |
| W18 | | tgaccttgtttgcttcctctg | caatagaccgtctgtgattgttg | 153 | 60°C |
| W19 | | cctcaacctctcatccactca | caagctacacaccagtgacca | 101 | 60°C |
| W20 | | aatgtgtgcacaatgaacatga | atatgctccatgctcgatga | 100 | 60°C |
| W21 | | tcgtctacggtcgctaagtg | ttcgtacagaaaaccacgtca | 109 | 60°C |
| W22 | | tctgtgtccttcaaatgatgc | aatgaggctgcacaatgaatc | 101 | 60°C |
| W24 | | tggagaggttggaaatctgg | caactctgaactgctatgcttca | 108 | 60°C |
| W25 | | atgccgtctcagtggtgatt | tgagacgcattcagtagtgc | 109 | 60°C |
| W26 | | tgaacatggttggttgattga | gcttccactataccgcctctc | 102 | 60°C |
| W27 | | caggaaatgcctcttcaagc | ttcatcatctcaactctcaacattact | 102 | 60°C |
| W28 | | cactgccttgtggattacacc | tttgtctgaggactggaataca | 108 | 60°C |
| W29 | | gtgagcacttaaacactagacca | acattggtggatgtgtcg | 97 | 55°C |
| W30 | | gtcattcgtacaataacaactta | ttgaaaagataacaactcagaac | 96 | 55°C |
| W31 | | cgaaaaacattttcatatacgtt | tcgccactaactcacaactaac | 92 | 55°C |
| W32 | | acaagtatacaaaggatagttttcg | tcaattatattcatctcatcaa | 89 | 55°C |
| W33 | | tagtgacttgataggaagttgt | tcaattatattcatctcatcaa | 86 | 55°C |
| W34 | | tttttataaacaattatgtagtgag | gcctctaaatcacaactagc | 72 | 55°C |
| W35 | | ttggatatgttcatcgactgt | tcaattatattcatctcatcaa | 80 | 55°C |
| qPCR for transcription of repeats and retrotransposons | | | | | |
| W1 | | ggagtggtgatgccagttcgagt | ggtccagcacaaccacacaccc | 111 | 60°C |
| W2 | | gcttgctgatgtgcagtttgccg | tgcgcaacgattctcggcact | 128 | 60°C |
| W3 | | aggaaaattacgaatgcg | ttcaccaggttgttcgtg | 135 | 60°C |
| W4 | | cggtcatggaggcgtggtca | ctcgtcgtaccatgacaatcagca | 130 | 60°C |
| W5 | | accgcgtctcccctcttcagt | agggagagcgcacaccatga | 138 | 60°C |
| SMAlphafem-1 | | tgcacaagtgagtggctgtggg | tggatgtacctgcatcccgtgt | 128 | 60°C |
| Perere-3 | | ttctccaacccactctgtcc | gacgtggatttggcacacct | 197 | 60°C |
| Saci-1 | | attacgacgctcaaccaacc | ccccgattgtacaagatgc | 224 | 60°C |
| Saci-5 | | atttcgggatcagctttcct | gcagaagtggacagcaacaa | 203 | 60°C |
| Saci-7 | | ggtggtgtcagtggcttttt | tacccggccaactagtcaac | 202 | 60°C |
| Reference loci | | | | | |
| Smp_scaff018821 | | gggcagacagtaacggcggc | tcgcgagaggtgcagtggga | 138 | 60°C |
| 28S | | gctgtagtggatctgtgc | ctacgtcatgggacgg | 400 | 60°C |
| aTub | | agcagttaagcgttgcagaaatca | tgacgagggtcacatttcaccta | 77 | 60°C |
| PCR for generation of FISH probes | | | | | |
| W1 | | aacacagtgaaattcttccttca | attcaactcaatgttcgaaatga | 456 | 42°C |
| W3 | | cggtcgtgttgaaggaaac | tggaaactgatgtgcgaaag | 678 | 42°C |
| W4 | | catggactgcaatacctttttaga | ctagttcgatattagtacatttatctttgc | 640 | 42°C |
| W5 | | atgtgaagatatggagagcga | aggatgccagacgaggatg | 1050 | 42°C |
| W6 | | agaaacttgactcaatagaatactgaaaa | cagctcaacaatctccacca | 283 | 42°C |
| W7 | | tcgaattgaaatgggtcacg | cagtctactcaccaacttctctatcc | 966 | 42°C |
| W8 | | atcatagttcaaattgtgcaataca | tctctcatcatccactcgtcg | 245 | 42°C |
| W13 | | tcatcattgtacattcacactcg | ttggttttgacttcagttgtgttt | 234 | 42°C |
| qPCR for confirmation of Z-specific sequences | | | | | |
| Smp_073450 | Smp_Scaff000252 | agacactttatgccagcga | tctttaactgaacgcttaggac | 134 | 60°C |
| Smp_171640 | Smp_Scaff000425 | gttccgtcgagaaatcgt | atggtgaagatgatgatggg | 157 | 60°C |
| Smp_136660 | Smp_Scaff000054 | atcgaccatcatcctctttag | gtgttgaccaatctgtccc | 101 | 60°C |
| Smp_128350 | Smp_Scaff000019 | accgatcagatttcattgtctc | gtcagcattcaagttatcgttc | 116 | 60°C |
| Smp_025100 | Smp_Scaff000050 | agtgaggtgtattgtggc | cgcattcaccatacgtc | 100 | 60°C |
| Smp_132060 | Smp_Scaff000034 | caaatcaacaaatcaatgtgatagca | ggctgatgaaagttgtatggg | 207 | 60°C |
| Smp_163870 | Smp_Scaff000264 | tacacgttgtcgtaatcatgg | ttcaacaacaagtaaacaagcag | 103 | 60°C |
| Smp_135000 | Smp_Scaff000047 | cttattactcaatgtttcccgt | ttcggctttgttgttatgatcta | 93 | 60°C |
| Smp_135140 | Smp_Scaff000047 | cctggtttaactatgggtttagg | atggctgggaaacagag | 91 | 60°C |
| Smp_140070 | Smp_Scaff000074 | cacatggtttgcagctttc | tctggtggacaagtaagtga | 88 | 60°C |
| Smp_140100 | Smp_Scaff000074 | ttacagcagacctgggatag | tacgttgcattctcattgac | 99 | 60°C |
| Smp_028440.1 | Smp_Scaff000059 | aggcgttacagaagaaactac | tccatccaccaacgattcac | 187 | 60°C |
| Smp_171710 | Smp_Scaff000425 | cgttagataatgctcgcca | gtctgcacgtaatcggt | 80 | 60°C |
| Smp_007650 | Smp_Scaff000012 | attctcgttctgcttattctct | gcattcaatatccgtccagtcta | 101 | 60°C |
| Smp_014170.2 | Smp_Scaff000024 | agcacctgttccgattg | agcattctgagaaggagtag | 112 | 60°C |
| Smp_147450 | Smp_Scaff000120 | atgagaatccgcatccat | aaacgagtcaatttatcttgctt | 114 | 60°C |
